# Bioinformatic screening for candidate biomarkers and their prognostic values in endometrial cancer

**DOI:** 10.1186/s12863-020-00898-4

**Published:** 2020-09-22

**Authors:** Yaowei Li, Li Li

**Affiliations:** 1 Department of Gynecologic Oncology, Affiliated Tumor Hospital of Guangxi Medical University, Key Laboratory of Early Prevention and Treatment for Regional High Frequency Tumor, Ministry of Education, Nanning, Guangxi 530021 People’s Republic of China; 2 Department of Gynecology and obstetrics, Shangyu People’s Hospital, Shangyu, Zhejiang 312300 People’s Republic of China

**Keywords:** Endometrial cancer, Differentially expressed genes, Differentially expressed miRNAs, Functional enrichment analysis, Protein-protein interaction, Survival analysis

## Abstract

**Background:**

Endometrial cancer is a common gynecological cancer with annually increasing incidence worldwide. However, the biomarkers that provide prognosis and progression for this disease remain elusive.

**Results:**

Two eligible human endometrial cancer datasets (GSE17025 and GSE25405) were selected for the study. A total of 520 differentially expressed mRNAs and 30 differentially expressed miRNAs were identified. These mRNAs were mainly enriched in cell cycle, skeletal system development, vasculature development, oocyte maturation, and oocyte meiosis signalling pathways. A total of 160 pairs of differentially expressed miRNAs and mRNAs, including 22 differentially expressed miRNAs and 71 overlapping differentially expressed mRNAs, were validated in endometrial cancer samples using starBase v2.0 project. The prognosis analysis revealed that Cyclin E1 (CCNE1, one of the 82 hub genes, which correlated with hsa-miR-195 and hsa-miR-424) was significantly linked to a worse overall survival in endometrial cancer patients.

**Conclusions:**

The hub genes and differentially expressed miRNAs identified in this study might be used as prognostic biomarkers for endometrial cancer and molecular targets for its treatment.

## Background

Endometrial cancer (EC), that is, uterine corpus endometrial carcinoma (UCEC), originates from the epithelial malignant tumours in endometrium. With an increase in obesity and an aging population, the incidence and mortality rates of EC are increasing in developed countries [1]. According to the latest statistics of the American Cancer Society [2], over 61, 000 cases were estimated to be diagnosed with EC in 2017. At present, advanced stage EC still accounts for 20–30% of incidents, and the disease relapse is associated with a poor prognosis.

Currently, there are no known reliable diagnostic and prognostic biomarkers for EC. Cancer antigen 125 (CA125), being most frequently used as a biomarker for ovarian cancer, has some diagnostic/prognostic value in EC [3]. However, CA125 level is elevated in a number of physiological and pathological conditions, such as age [4, 5], pregnancy [6], menstruation [4, 6], and in gynaecological and non-gynaecological disorders, such as endometriosis [6], benign ovarian cysts [6], pelvic inflammatory disease [6], peritonitis [6], pancreatitis [6], and pneumonia [6]. Human epididymis protein 4 (HE4) also has some diagnosis/prognosis value in EC [7]. Similar to the high expression of CA125, HE4 level is also elevated in many physiological and non-gynaecological conditions, such as age [8], menopausal status [8], Body Mass Index [8], smoking status [8], creatine levels [8], pulmonary adenocarcinoma [9], chronic kidney disease [7], renal failure [10], and kidney fibrosis [11].

Due to these factors reduce the clinical value of the existing biomarkers in the progress and prognosis of EC, it is crucial to discover new reliable biomarkers as well as to unravel the underlying molecular mechanisms of the EC progression.

## Results

### Identification of DEGs and DEMs

**Fig. 1:**
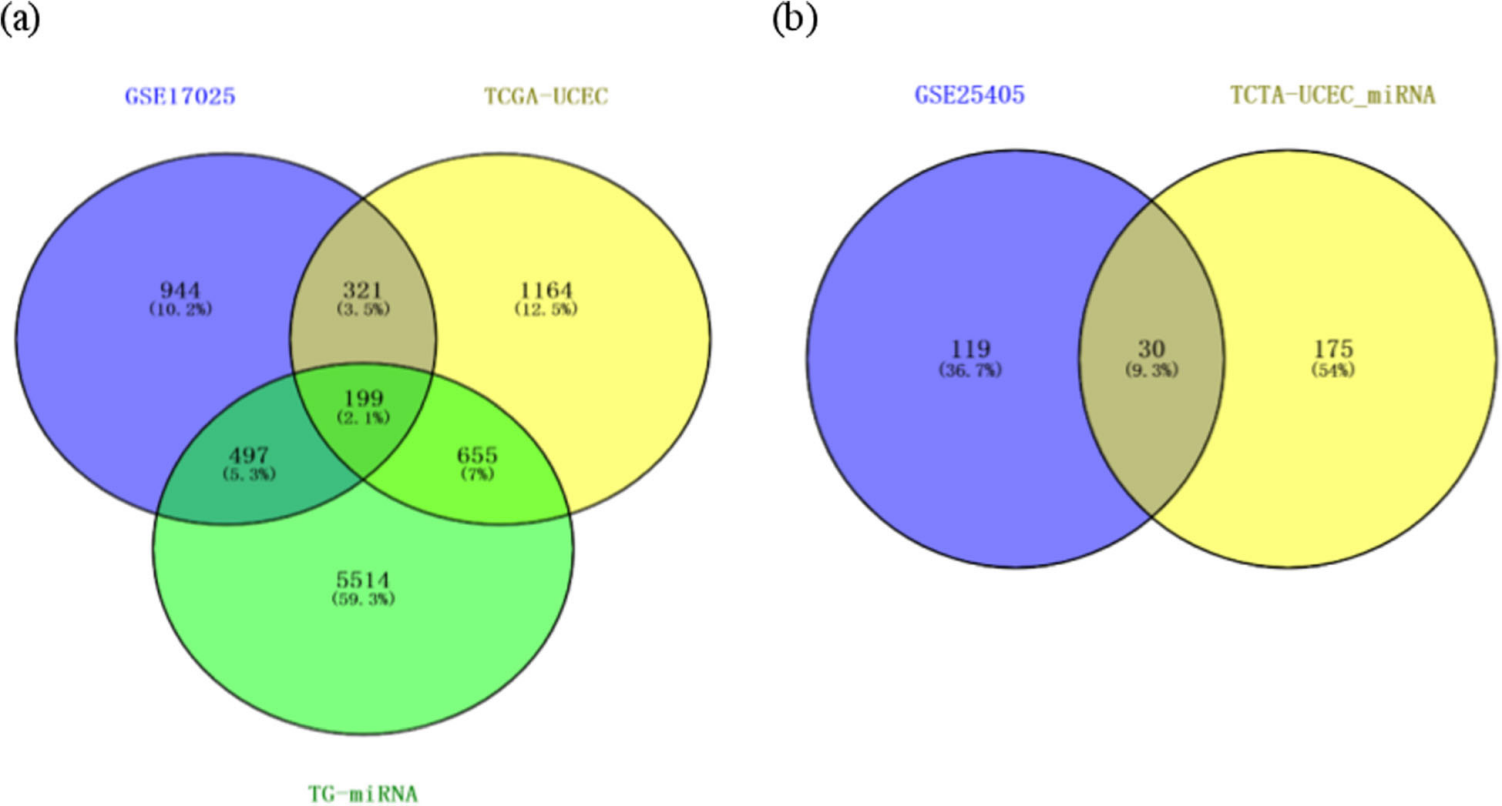
**a**: Venn diagram of the differentially expressed genes among these three datasets. **b**: Venn diagram of the differentially expressed miRNAs between two datasets. TCGA-UCEC: the mRNA data of uterine corpus endometrial carcinoma in the Cancer Genome Atlas, TCGA-UCEC_miRNA: the miRNA data of uterine corpus endometrial carcinoma in the Cancer Genome Atlas, TG-miRNA: the target gene of differentially expressed miRNA

A total of 1961 DEGs and 149 DEMs were identified from GSE17025 and GSE25405, respectively; 2339 DEGs and 205 DEMs were identified from the mRNA and miRNA data of uterine corpus endometrial carcinoma in TCGA (named TCGA-UCEC and TCGA-UCEC_miRNA, respectively); 520 common DEGs and 30 common DEMs were screened out with Venny 2.1.0(http://bioinfogp.cnb.csic.es/tools/venny/index.html) [12], respectively (Fig. 1a, Fig. 1b). There were 212 upregulated genes and 308 downregulated genes, as well as 15 upregulated and 15 downregulated miRNAs in EC tissues compared with NE tissues, respectively (Table 1, Table 2).

**Table 1:** Top 10 DEGs in EC tissues compared with NE tissues according to the data from TCGA database

DEG	logFC	*P*-value	adj. *P*-value
upregulated genes			
SFN	4.465258403	5.76E-42	2.15E-40
PRAME	4.193768196	5.79E-69	7.66E-67
MYBL2	4.109297769	2.84E-74	4.67E-72
UBE2C	4.074765041	1.71E-69	2.33E-67
CDC20	3.919322957	1.25E-78	2.67E-76
AQP5	3.427650505	2.06E-18	2.18E-17
PRSS8	3.413404932	1.96E-49	1.06E-47
TK1	3.400033651	3.60E-77	6.98E-75
PI3	3.370250122	7.80E-14	5.87E-13
TPX2	3.308872927	1.19E-58	9.97E-57
downregulated genes			
DES	−6.619094047	8.36E-54	5.54E-52
MYH11	−5.635290069	6.17E-75	1.05E-72
CNN1	− 5.55734654	5.08E-64	5.45E-62
ACTG2	−4.902138932	4.13E-50	2.32E-48
LMOD1	−4.795641474	4.82E-86	1.42E-83
OGN	−4.663558704	9.95E-120	1.55E-116
DPT	−4.427179281	6.84E-99	3.98E-96
SPARCL1	−4.386222474	1.33E-70	1.90E-68
ZCCHC12	−4.328794652	2.64E-68	3.40E-66
SFRP4	−4.266562157	4.62E-26	7.95E-25

*DEGs* differentially expressed genes, *EC* endometrial cancer, *NE* normal endometrium, *FC* fold-change; adj. *P*-value, adjusted *P*-value; adjusted *P*-value was obtained by correcting *P*-value using the ‘Benjamini-Horchberg’ method

**Table 2:** Top 10 DEMs in EC tissues compared with NE tissues according to the data from GEO database

miRNA	logFC	*P*-value	adj. *P*-value
upregulated miRNA			
hsa-miR-205	5.717071419	8.28E-10	3.45E-07
hsa-miR-135b	3.002833732	6.43E-05	1.87E-03
hsa-miR-182	2.827333932	2.90E-06	2.00E-04
hsa-miR-183	2.705891835	2.87E-05	1.06E-03
hsa-miR-429	2.437603498	9.52E-07	9.11E-05
hsa-miR-200b	2.244848862	1.74E-06	1.37E-04
hsa-miR-96	2.195969063	7.78E-05	2.14E-03
hsa-miR-200a	1.973216466	4.25E-05	1.43E-03
hsa-miR-202	1.943991213	4.19E-03	4.31E-02
hsa-miR-210	1.715073494	1.94E-04	3.85E-03
downregulated miRNA			
hsa-miR-424	−4.875026249	1.77E-13	3.59E-10
hsa-miR-143	−4.140227835	5.97E-07	6.07E-05
hsa-miR-133b	−4.081321667	4.19E-06	2.54E-04
hsa-miR-376c	−3.636752313	9.60E-05	2.41E-03
hsa-miR-195	−3.523216268	1.67E-07	2.57E-05
hsa-miR-204	−3.51217031	7.22E-04	1.10E-02
hsa-miR-145	−3.493087645	1.82E-05	7.61E-04
hsa-miR-411	−3.39771373	3.84E-06	2.40E-04
hsa-miR-381	−3.035325968	4.20E-05	1.41E-03
hsa-miR-379	−2.971031318	2.79E-06	1.95E-04

*DEMs*, differentially expressed miRNAs, *EC* endometrial cancer, *NE* normal endometrium, *miRNA or miR*, microRNA, *FC*, fold-change; adj. *P*-value, adjusted *P*-value; adjusted *P*-value was obtained by correcting P-value using the ‘Benjamini-Horchberg’ method

### Functional and pathway enrichment analysis

The functional and pathway enrichment analyses of DEGs were conducted with DAVID. The upregulated genes were mainly enriched in these biological processes, which were cell cycle, cell division, and DNA replication signalling pathways, while downregulated genes were mainly enriched in skeletal system development, vasculature development, and cell adhesion signalling pathways (Table 3). Moreover, three KEGG pathways were enriched in upregulated genes, including cell cycle, oocyte maturation, and oocyte meiosis signalling pathways (Table 3). There were no KEGG pathways enriched in downregulated genes.

**Table 3:** Top 10 GO terms of biological processes and significant KEGG pathways of upregulated and downregulated DEGs for EC tissues compared with NE tissues

Term	Description	Count	*P*-Value	FDR
Upregulated DEGs				
GO:0022403	cell cycle phase	47	1.16E-30	1.93E-27
GO:0000279	M phase	42	2.96E-29	4.94E-26
GO:0000278	mitotic cell cycle	42	3.27E-27	5.44E-24
GO:0000280	nuclear division	34	3.43E-26	5.71E-23
GO:0007067	mitosis	34	3.43E-26	5.71E-23
GO:0000087	M phase of mitotic cell cycle	34	6.25E-26	1.04E-22
GO:0022402	cell cycle process	48	9.63E-26	1.60E-22
GO:0048285	organelle fission	34	1.30E-25	2.16E-22
GO:0007049	cell cycle	53	3.71E-24	6.18E-21
GO:0051301	cell division	33	5.63E-21	9.38E-18
KEGG pathway				
hsa04110	Cell cycle	18	1.70E-11	1.87E-08
hsa04914	Progesterone-mediated oocyte maturation	10	1.30E-05	1.42E-02
hsa04114	oocyte meiosis	11	1.48E-05	1.62E-02
Downregulated DEGs				
GO:0001501	skeletal system development	21	2.58E-07	4.35E-04
GO:0001944	vasculature development	16	1.53E-05	2.57E-02
GO:0007155	cell adhesion	28	2.67E-05	4.50E-02
GO:0022610	biological adhesion	28	2.74E-05	4.61E-02

*GO* gene ontology, *KEGG* Kyoto Encyclopedia of Genes and Genomes, *DEGs* differentially expressed genes, *EC* endometrial cancer, *NE* normal endometrium, *FDR* false discovery rate

### Construction of PPI network and module analysis

**Fig. 2:**
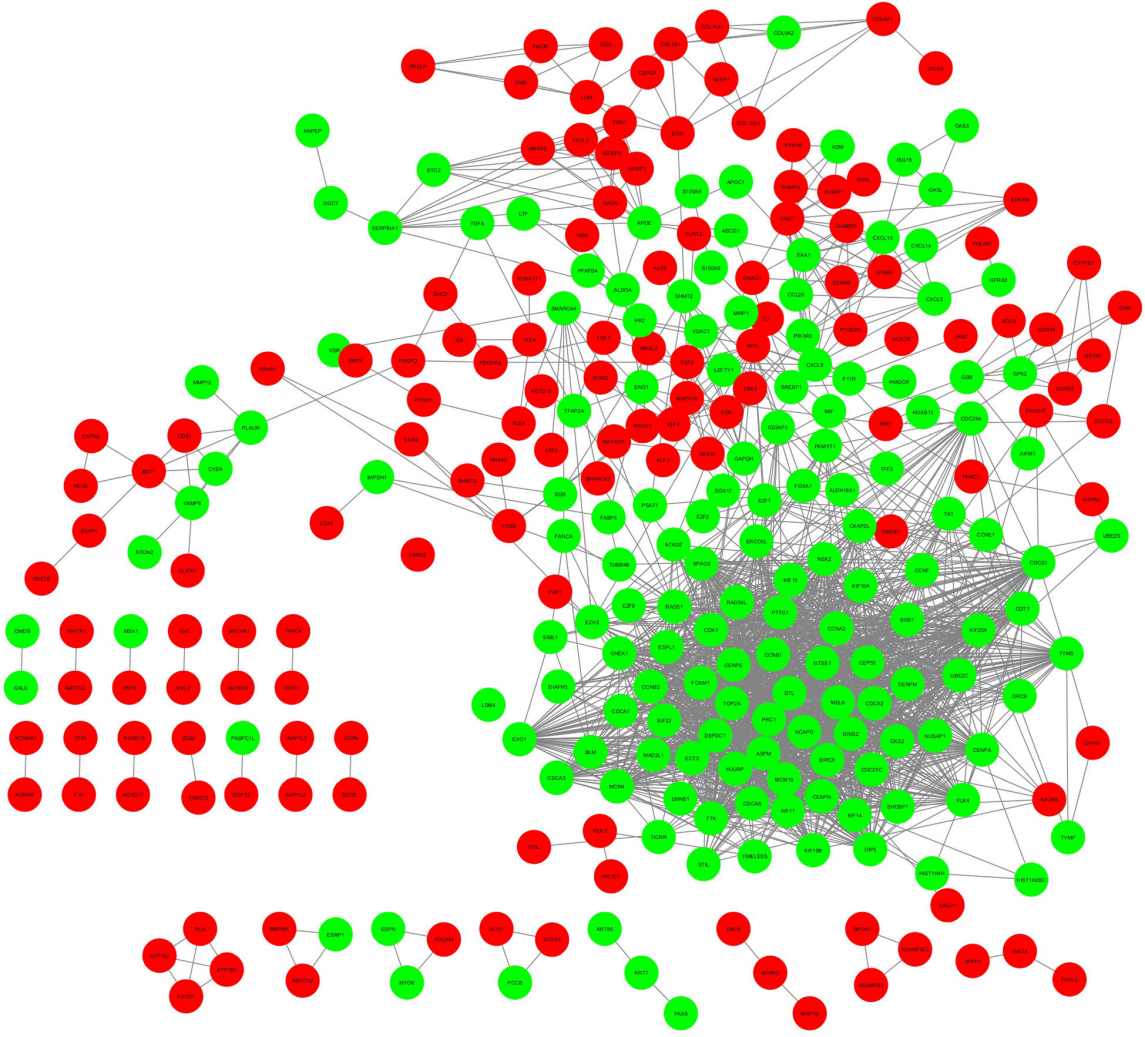
Protein-protein interaction network of the differentially expressed genes in endometrial cancer tissues compared with normal endometrium tissues. Green and red nodes represent upregulated and downregulated genes, respectively. The edges/lines stand for the regulatory association between nodes

**Fig. 3:**
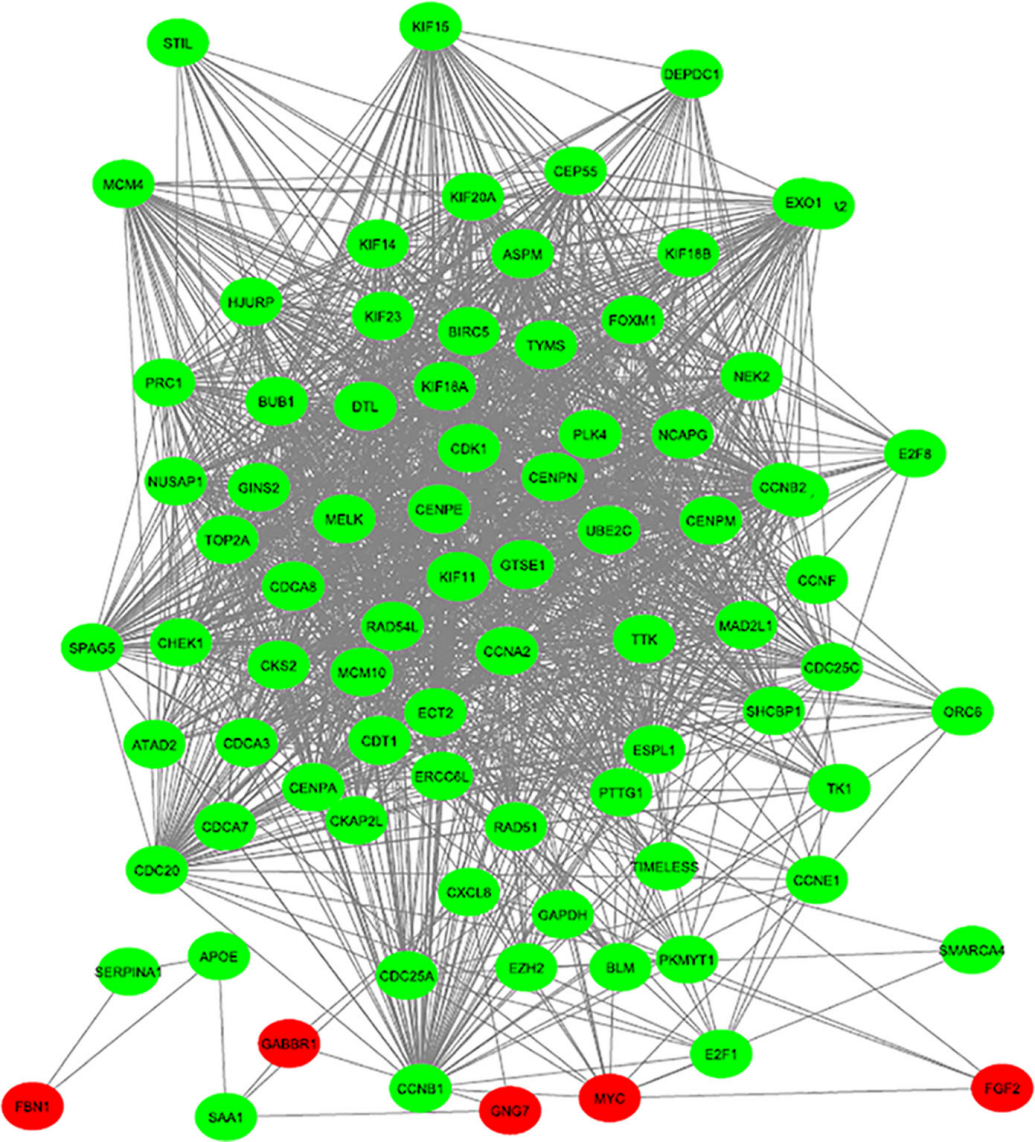
Protein-protein interaction network of hub genes of the differentially expressed genes in endometrial cancer tissues compared with normal endometrium tissues. Green and red nodes represent upregulated and downregulated genes, respectively. The edges/lines stand for the regulatory association between nodes

**Fig. 4:**
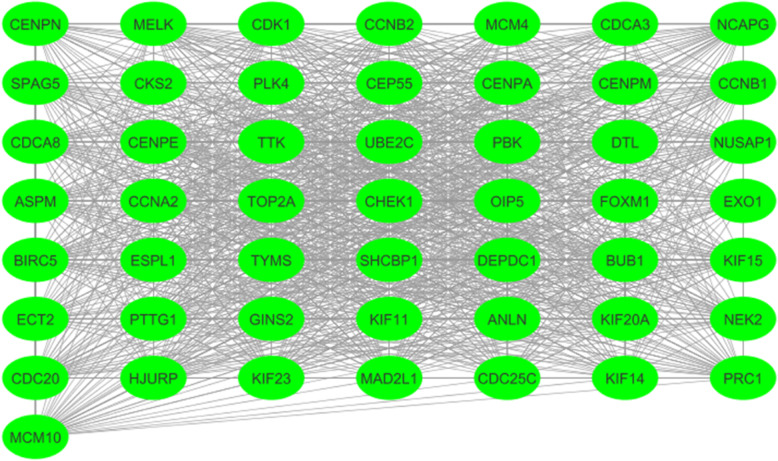
Demonstration of the important module by cytoscape. The edges/lines stand for interaction relationship between nodes

A PPI network consisting of 287 nodes and 1840 edges was constructed, which included 212 upregulated and 308 downregulated genes (Fig. 2). Next, 82 genes were screened out as hub genes (Degree of interaction ≥10 were selected as the threshold) [13], there were close correlations among hub genes (Fig. 3, Additional file 1). After analysing the network with the MCODE tool in Cytoscape software, an important module was obtained, including 50 nodes and 1082 edges (Fig. 4). Functional enrichment analyses of biological processes with regard to this module showed that these genes were enriched in cell cycle, cell division, and DNA replication signalling pathways (Table 4). Three KEGG analysis showed an enrichment in cell cycle, oocyte meiosis, and oocyte maturation signalling pathways (Table 4).

**Table 4:** Top 10 GO terms of biological processes and significant KEGG pathways of the DEGs in module

Term	Description	Count	*P*-value	FDR
Biological processes				
GO:0000279	M phase	32	8.12E-40	1.14E-36
GO:0022403	cell cycle phase	32	1.24E-36	1.75E-33
GO:0000278	mitotic cell cycle	31	2.61E-36	3.67E-33
GO:0000280	nuclear division	27	2.86E-35	4.02E-32
GO:0007067	mitosis	27	2.86E-35	4.02E-32
GO:0000087	M phase of mitotic cell cycle	27	4.67E-35	6.57E-32
GO:0048285	organelle fission	27	8.52E-35	1.20E-31
GO:0022402	cell cycle process	33	4.25E-34	5.97E-31
GO:0007049	cell cycle	35	6.14E-33	8.63E-30
GO:0051301	cell division	27	7.85E-32	1.10E-28
KEGG pathway				
hsa04110	Cell cycle	13	4.76E-17	2.96E-14
hsa04114	oocyte meiosis	9	4.14E-10	2.58E-07
hsa04914	Progesterone-mediated oocyte maturation	7	1.37E-07	8.55E-05

*GO* gene ontology, *KEGG* Kyoto Encyclopedia of Genes and Genomes, *DEGs* differentially expressed genes, *FDR* false discovery rate

### Analysis of miRNA-mRNA regulatory network

**Fig. 5:**
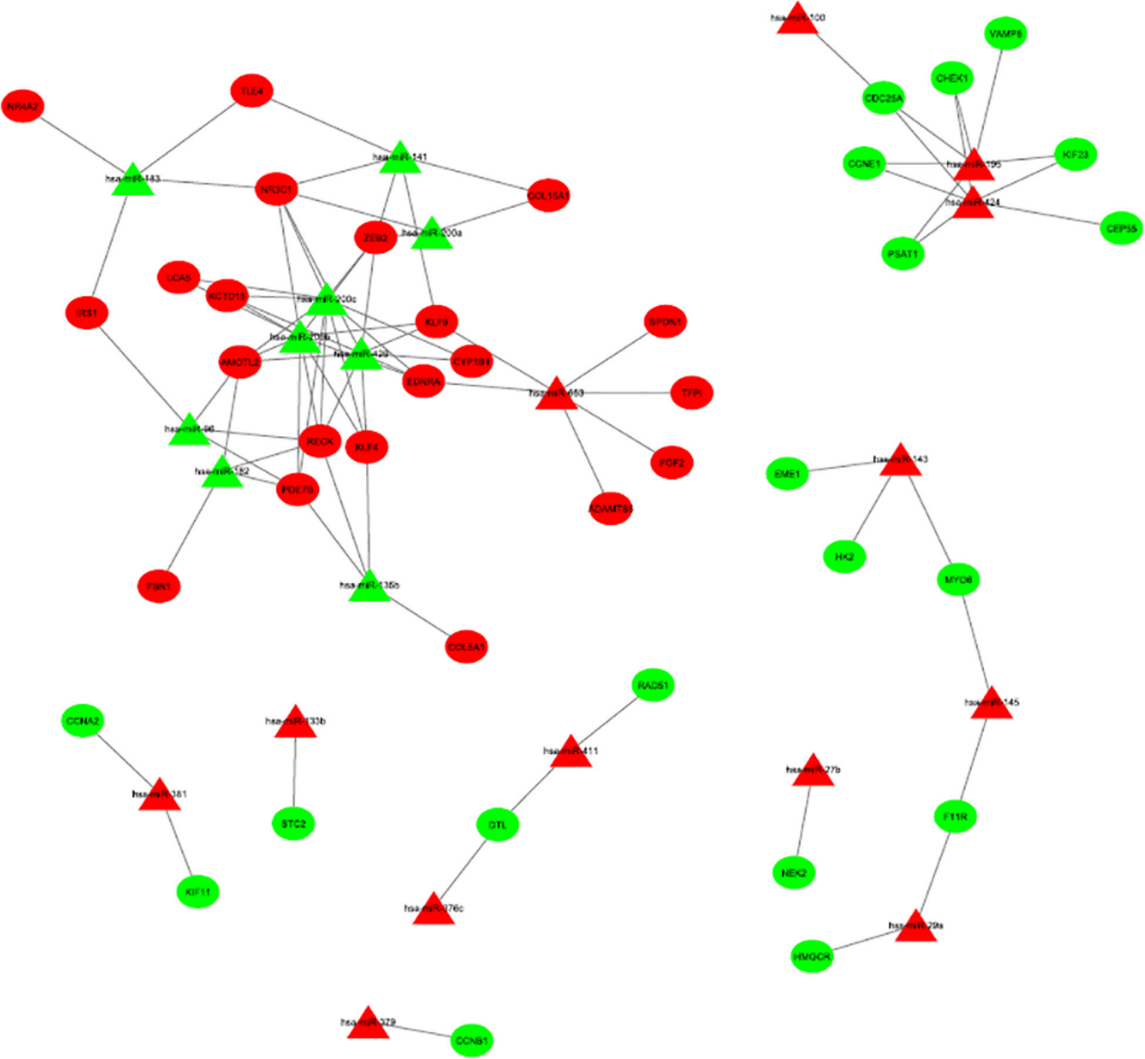
The miRNA-mRNA regulatory network. Green and red nodes stand for upregulation and downregulation, respectively. The ellipses represent genes and the triangles represent miRNAs

Thirty commonly identified DEMs were screened out from GSE25405 and TCGA-UCEC_miRNA, including 15 upregulated and 15 downregulated miRNAs (Table 2). 6865 TG-miRNAs were checked in the miRecords database, of which 199 were validated in 520 common DEGs (Fig. 1a). These 199 commonly identified DEGs and 30 commonly identified DEMs were used to construct a miRNA-mRNA network. In patients with EC, 160 pairs of DEMs-DEGs relationships with reverse associated expression were confirmed using starBase v2.0 project, including 22 DEMs and 71 overlapping DEGs (Fig. 5, Additional file 2). In the network, hsa-miR-200b, hsa-miR-200c, hsa-miR-429, hsa-miR-424, hsa-miR-195, hsa-miR-653, and hsa-miR-141 showed a higher degree of interaction (≥ 5, Table 5).

**Table 5:** Top 7 miRNAs with the highest degree of interaction in the miRNA-mRNA interactions network (Degree of interaction ≥5)

Node	Degree of interaction
hsa-miR-200b	10
hsa-miR-200c	10
hsa-miR-429	9
hsa-miR-424	6
hsa-miR-195	6
hsa-miR-653	6
hsa-miR-141	5

*miR* microRNA

### Survival analysis

**Fig. 6:**
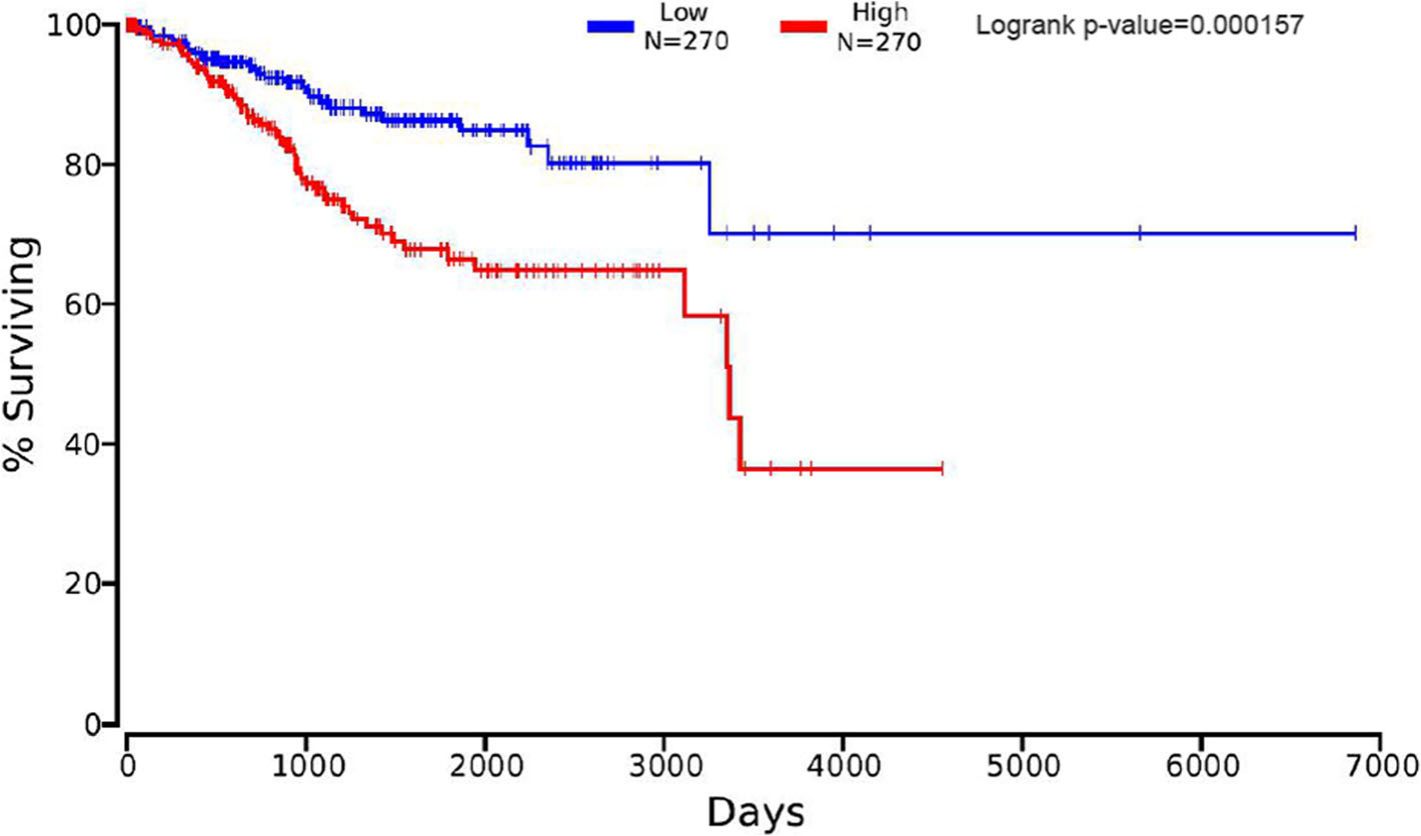
Overall survival analysis of CCNE1 expression with prognosis of endometrial cancer patients (Logrank *p*-value = 0.000157). Based on the median expression level of CCNE1, the patients with EC were divided into two (high vs. low) groups

**Fig. 7:**
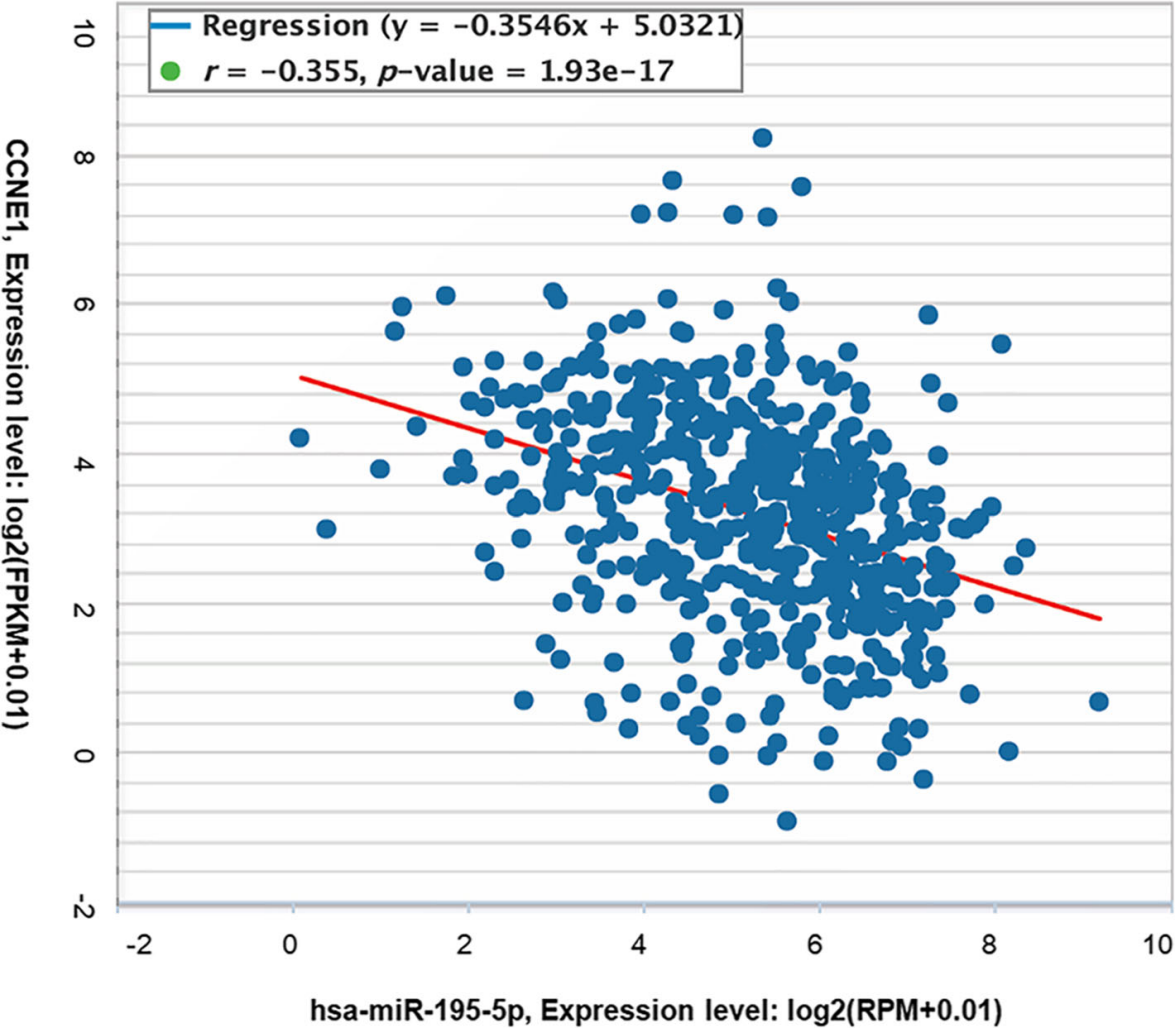
The correlated expression of CCNE1 and hsa-miR-195-5p (hsa-miR-195) in 538 patients with endometrial cancer. The correlation coefficients − 0.355 with p-value = 1.93e-17 indicated that CCNE1 and hsa-miR-195 expression levels were correlated with each other; data source: starBase v3.0 project

The prognostic value of 82 hub genes was assessed by OncoLnc. We found that high mRNA expression of BUB1, TOP2A, CDCA8, TTK, ASPM, UBE2C, BIRC5, HJURP, CENPA, MCM10, FOXM1, SPAG5, EXO1, ESPL1, OIP5, MCM4, CDC25C, DEPDC1, KIF18B, ERCC6L, CKAP2L, ATAD2, TK1, CCNF, E2F1, and CCNE1, as well as low mRNA expression of MYC was associated with the significantly worse overall survival for EC patients (data not shown). What makes us interesting was that, CCNE1 was also identified as a target gene of hsa-miR-195 and hsa-miR-424, which were identified in our DEM analysis (Fig. 6, Fig. 7).

## Discussion

In recent years, although clinical medical scientists have made significant progress in the treatment of EC with surgery and chemotherapy, the incidence and mortality rate of EC is still rising [14]. It is necessary to further understand the etiology and underlying mechanism of the EC progression to improve the prognosis of EC.

In this study, by integrating GSE17025 and TCGA-UCEC datasets, 520 common DEGs were screened out in EC tissues compared with NE tissues. These 520 common DEGs were composed of 212 upregulated genes and 308 downregulated genes. The upregulated DEGs, such signalling pathways, were mainly enriched as cell cycle, cell division, and DNA replication. Skeletal system development, vasculature development, and cell adhesion signalling pathways were enriched among downregulated DEGs. Furthermore, PPI network was built for 82 hub genes. Survival association analysis of these 82 hub genes showed poor prognosis associated with 26 upregulated genes and one downregulated gene for patients with EC. Similarly, 30 common DEMs were analysed from GSE25405 and TCGA-UCEC_miRNA datasets. After integrating 6865 TG-miRNAs with these 520 common DEGs, 71 overlapping DEGs were screened that showed close correlations with 22 common DEMs in EC (Fig. 5, Additional file 2). Moreover, high mRNA expression of CCNE1 (one of the 82 hub genes, which was correlated with hsa-miR-195 and hsa-miR-424) was significantly correlated with worse overall survival in EC patients.

miRNAs are endogenous small non-coding RNAs, which can inhibit gene expression by mRNA degradation/destabilization or through impairing translation [15, 16]. The abnormal expression of miRNAs occurs in a variety of tumours and is often associated with altered tumour characteristics, such as changes in tumour cell survival, proliferation, and invasion [17].

In this study, 30 common DEMs were compared between EC and NE tissues, such as hsa-miR-200b, hsa-miR-200c, hsa-miR-429, hsa-miR-141, hsa-miR-424, hsa-miR-195, and hsa-miR-653. The microRNA-200 (miR-200) family consists of miR-200a, miR-200b, miR-200c, miR-429, and miR-141, which all have the same seed sequence and homologous targets. The expression of hsa-miR-200b is upregulated in many malignant tumours [18–20], and its role in the inhibition of mesenchymal characteristics and metastasis has been revealed in prostate cancer, gastric carcinoma, and hepatocellular carcinoma via regulating ZEB1 expression or directly targeting ZEB2, or via Rho/ROCK signalling pathway [21–23]. Our study outcomes suggested that hsa-miR-200b was also upregulated in EC, and the observation was consistent with the previous study [24]. Hsa-miR-200c has been widely investigated during the last few years. There have been numerous studies demonstrating the association between an aberrant expression level of miR-200c and the prognosis of various human malignancies, such as breast cancer [18, 25, 26], prostate cancer [27], ovarian cancer [28], and endometrial cancer [29]. Some of these studies verified the anti-oncogenic role of miR-200c in certain cancer types, indicating the potential correlation of elevated expression levels of miR-200c and superior prognosis [26, 28, 29]. In contrast, other studies have suggested that miR-200c serves as an oncogene [18, 25, 27]. Nevertheless, these findings suggest that miR-200c is a potential biomarker for cancer prognosis. Our results also suggested that hsa-miR-200c was upregulated, and the observation was consistent with the previous study [29]. Recent reports have shown that hsa-miR-429 expression is frequently upregulated in several cancers and may function as an oncogene [30, 31] in cancers, such as endometrial carcinoma [30], as observed in this study. One study showed that upregulation of hsa-miR-429 is associated with a decrease in overall survival of serous ovarian cancer [32]; in contrast, other studies have shown that hsa-miR-429 was downregulated in some malignant tumours and had tumour-suppressor function [33, 34]. These results indicate that hsa-miR-429 plays different (even opposite) roles in tumorigenesis and cancer progression in different tumours. Hsa-miR-141 is also an important member of the miR-200 family, several previous studies have shown that has-miR-141 was involved in prognosis of cancer [35–37].

Some previous studies reported that hsa-miR-424 was downregulated and could have a tumour suppressor role in some cancers [38–40]. In line with these observations, our present study also showed that hsa-miR-424 was downregulated [40]. Hsa-miR-195 is a member of the miR-15a, −15b, − 16, − 195, − 424, and − 497 families, which is involved in the occurrence and developmental progress of many malignant tumours and regulation of malignant biological behaviours [40–43]. In our study, hsa-miR-195 in EC tissues showed lower expression levels compared with NE tissues, which was consistent with the previous study [42]. So far, there are only few reports on the role of hsa-miR-653 in the malignant biological behaviour of tumours.

Based on our findings, we speculates that hsa-miR-200b, hsa-miR-200c, hsa-miR-429, hsa-miR-141, hsa-miR-424, hsa-miR-195, and hsa-miR-653 may play important roles in biological behavior of EC by multiple pathways.

CCNE1, that is Cyclin E1, belongs to the cyclin family which, through association with cyclin-dependent kinase 2, controls cell cycle progression from G1 to S phase [44]. Previous studies have shown that the upregulation of CCNE1 could contribute to cancer development or tumorigenesis in many cancers [45–50], and CCNE1 could serve as a reliable independent prognostic marker [49, 50]. miRNAs from multiple families have been identified to target CCNE1 in a number of malignant tumours, such as hepatocellular carcinoma [51], osteosarcoma [52], cervical cancer [53], and bladder cancer [54]. In the present study, survival analysis of the hub genes related to DEMs showed that high expression of CCNE1 could indicate poor prognosis in EC patients.

There are some defects in this article. Such as, the overlapped miRNAs were about only 1/4 to 1/5 between GSE25405 and TCGA-miRNA, and some of the findings need further experimental validation in future studies.

With regard to the ratio of the overlapped miRNAs is low, the following observations may explain the possible reasons. Firstly, the ethnic origins of the chip and RNA-seq samples were different. The GSE25405 data was composed of Asians, while the TCGA-miRNA data was mainly composed of European Americans and African Americans. Secondly, the sample sizes were also different; while GSE25405 included 48 samples (41 endometrial cancer tissue samples, 7 normal endometrial tissue samples), the TCGA data sample size was larger and (after the author has screened and processed the relevant data) a total of 572 samples were included (539 tissue samples from endometrial cancer patients and 33 normal controls). Last but not least, the efficacy of RNA-seq detection and chip detection were different. It is well known that when detecting genes with higher abundance, the results of RNA-seq and chip may be similar, however, when detecting genes with lower abundance, RNA-seq can more effectively capture relevant information. As for the latter topic, we believe that the outcomes of the present study provide credible base for future research. For example, verifying the expression of selected miRNA (such as, miR-195 and miR-424.) in endometrial cancer cell lines and endometrial cancer tissue samples through PCR experiments, and in animal models may shed light on the role of these miRNAs in affecting the malignant biological process of endometrial cancer. Further, verifying the differential expression of miRNA in a large number of clinical samples and to analyse its correlation with clinical parameters (such as tumour clinical stage, pathological stage classification, recurrence, metastasis, and prognosis.) will help to determine the diagnosis and prognostic value of these miRNA in endometrial cancer patients. For another example, one or more hub genes can be selected to verify their mRNA and protein expression in endometrial cancer cell lines and endometrial cancer tissue samples. And then study the effect of genes, which were knocked out or overexpressed or mutated, on the biological process of endometrial cancer cell lines (such as, tumour cell proliferation, transformation, migration and invasion, blood vessel formation, and energy metabolism.) and its participation in molecular mechanism of action / signal transmission research. What’s more, to establish a subcutaneous transplanted tumour model, to introduce the target gene into the animal body, to observe the effect on tumour growth in the body, and further analyse the molecular mechanism or signal transmission of the target gene to provide potential targets for tumour gene therapy. Lastly, to verify the different expression of genes in a large number of clinical samples and analyse its correlation with clinical parameters to determine the diagnosis and prognostic value of genes in endometrial cancer patients.

Next, our clinical research team will select some miRNAs to verify the relationship between miRNAs and target genes through clinical experiments and their value in the diagnosis and prognosis of endometrial cancer patients.

## Conclusions

Based on bioinformatics analyses of EC-related microarray data in the GEO database and clinical data related to EC in TCGA database, we identified 27 hub genes (BUB1, TOP2A, CDCA8, TTK, ASPM, UBE2C, BIRC5, HJURP, CENPA, MCM10, FOXM1, SPAG5, EXO1, ESPL1, OIP5, MCM4, CDC25C, DEPDC1, KIF18B, ERCC6L, CKAP2L, ATAD2, TK1, CCNF, E2F1, CCNE1, and MYC) that were associated with poor prognosis in EC patients. Further, seven miRNAs (hsa-miR-200b, hsa-miR-200c, hsa-miR-429, hsa-miR-141, hsa-miR-424, hsa-miR-195, and hsa-miR-653) were observed to participate in biological behaviour of EC. Further research is warranted to confirm the clinical implications of our findings.

## Methods

### Microarray expression data

The mRNA and miRNA expression data of the GSE17025 and GSE25405 datasets were respectively downloaded from the GEO database (https://www.ncbi.nlm.nih.gov/geo/). The mRNA dataset GSE17025 contained the data from 103 samples, including 91 EC tissue samples and 12 normal endometrium (NE) samples. mRNA expression profiles in this dataset were measured using the GPL570 [HG.U133_Plus_2] Affymetrix Human Genome U133 Plus 2.0 Array platform [55]. The miRNA dataset GSE25405 contained 41 EC tissue samples and 7 NE tissue samples. In this dataset, the miRNA expression profile was detected using the GPL7731 Agilent-019118 Human miRNA Microarray 2.0 G4470B platform.

### The RNA-seq data

The mRNA-seq and miRNA-seq data of patients with UCEC were downloaded from TCGA (www.cancergenome.nih.gov) by the tool named SangerBox (https://shengxin.ren/softs/Sanger_V1.0.8.zip; accessed June 20, 2019). The mRNA-seq and miRNA-seq datasets contained 544 EC tissue samples, 35 NE tissue samples, and 539 EC tissue samples and 33 NE tissue samples, respectively.

### Identification of DEGs and DEMs

The Limma package (version 3.36.5) in R/Bioconductor was used to identify differentially expressed genes (DEGs) and differentially expressed miRNAs (DEMs) between EC and NE tissue samples [56]. The adjusted *P*-value (adj.P-value) was obtained by correcting P-value using the ‘Benjamini-Hochberg’ method, adj.P-value < 0.05 and |log_2_ fold change (FC)| > 1 were set as the threshold value [57]. The original probe-level data in Series Matrix Files were converted into gene symbol based on platform annotation files. The expression values of multiple probes corresponding to the same gene were selected by the minimum adj.P-value.

### Functional and pathway enrichment analysis

The Database for Annotation, Visualization and Integrated Discovery (DAVID, http://david.ncifcrf.gov) facilitates users to perform biological analysis from data collection [58]. Gene Ontology (GO) and Kyoto Encyclopedia of Genes and Genomes (KEGG) pathway enrichment analyses were conducted with DAVID. FDR < 0.05 was set as statistically significant.

### Construction of PPI network and module analysis

PPI network of DEGs was constructed using STRING database (version 11.0, https://string-db.org/) and visualized using Cytoscape (version 3.7.1) [59, 60]. The parameter was set as medium confidence score ≥ 0.7, module analyses were conducted using Cytoscape software MCODE package with degree cut-off = 2, node score cut-off = 0.2, max depth = 100 and k-score = 2 [61]. The functional enrichment analyses for these DEGs in the modules were conducted with DAVID.

### Prediction of the target gene of miRNA

The target genes for miRNAs (TG-miRNAs) were predicted by employing miRecords (http://c1.accurascience.com/miRecords/), which includes 11 different miRNA target genes predicted databases [62]. A TG-miRNA can only be identified when at least four different prediction databases predict that the gene is a target gene.

### Construction of the miRNA-mRNA regulatory network

The intersection of TG-miRNAs and DEGs were considered to be potentially valuable differentially expressed target genes. Pearson correlation analysis was then used in starBase (http://starbase.sysu.edu.cn/) to verify the association between these potentially valuable differentially expressed target genes and DEMs in patients with EC [63]. These significant differentially expression target genes and corresponding miRNAs were used to construct a miRNA-mRNA regulatory network using the Cytoscape software. The Degree of interaction of the node ≥5, which was defined as a hub miRNA.

### Survival analysis of hub genes

The overall survival of patients with EC with regard to hub genes was calculated using Kaplan-Meier analysis in OncoLnc (http://www.oncolnc.org/) [64]. The patients were divided into two groups (high vs. low) according to the median values of mRNA expression of the hub gene. The log-rank test was used to examine the significance of the difference between two groups.

## Supplementary information

**Additional file 1.** Node-degree of interaction analysis of the 82 hub genes (Degree of interaction ≥10).

**Additional file 2.** Correlation between differentially expressed miRNAs and target genes in patients with endometrial cancer (Data source: starBase v2.0 project).

## Data Availability

The mRNA and miRNA expression data of the GSE17025 and GSE25405 datasets can be respectively downloaded from the GEO database (GEO Accessions: GSE17025; GSE25405). The mRNA-seq and miRNA-seq data of patients with UCEC were directly downloaded from TCGA by the tool named SangerBox (https://shengxin.ren/softs/Sanger_V1.0.8.zip) without logging in the accession numbers or visiting website of TCGA.

## Abbreviations

EC: Endometrial cancer
UCEC: Uterine corpus endometrial carcinoma
CA125: Cancer antigen 125
HE4: Human epididymis protein 4
NE: Normal endometrium
DEGs: Differentially expressed genes
DEMs: Differentially expressed miRNAs
DAVID: Database for Annotation, Visualization and Integrated Discovery
GO: Gene Ontology
KEGG: Kyoto Encyclopedia of Genes and Genomes
PPI: Protein-protein interaction
TG-miRNAs: target genes for miRNAs

## References

[1] Lortet-Tieulent J, Ferlay J, Bray F, Jemal A. International patterns and trends in endometrial Cancer incidence, 1978-2013. J Natl Cancer Inst. 2018;110(4):354–61. 10.1093/jnci/djx214 29045681

[2] Siegel RL, Miller KD, Jemal A (2017). Cancer statistics, 2017. CA Cancer J Clin.

[3] Fambrini M, Sorbi F, Sisti G, Cioni R, Turrini I, Taddei G (2014). Endometrial carcinoma in high-risk populations: is it time to consider a screening policy?. Cytopathology.

[4] Hirsch M, Duffy J, Davis CJ, Nieves Plana M, Khan KS (2016). International collaboration to harmonise O, et al. diagnostic accuracy of cancer antigen 125 for endometriosis: a systematic review and meta-analysis. BJOG.

[5] Jiang T, Huang L, Zhang S (2015). Preoperative serum CA125: a useful marker for surgical management of endometrial cancer. BMC Cancer.

[6] Simmons AR, Baggerly K, Bast RC (2013). The emerging role of HE4 in the evaluation of epithelial ovarian and endometrial carcinomas. Oncology..

[7] Aggarwal P, Kehoe S (2010). Serum tumour markers in gynaecological cancers. Maturitas..

[8] Chen Y, Ren YL, Li N, Yi XF, Wang HY (2016). Serum human epididymis protein 4 vs. carbohydrate antigen 125 and their combination for endometrial cancer diagnosis: a meta-analysis. Eur Rev Med Pharmacol Sci.

[9] Bolstad N, Oijordsbakken M, Nustad K, Bjerner J (2012). Human epididymis protein 4 reference limits and natural variation in a Nordic reference population. Tumour Biol.

[10] Kamei M, Yamashita S, Tokuishi K, Hashioto T, Moroga T, Suehiro S (2010). HE4 expression can be associated with lymph node metastases and disease-free survival in breast cancer. Anticancer Res.

[11] Hertlein L, Stieber P, Kirschenhofer A, Krocker K, Nagel D, Lenhard M (2012). Human epididymis protein 4 (HE4) in benign and malignant diseases. Clin Chem Lab Med.

[12] Oliveros, J.C. (2007-2015) Venny. An interactive tool for comparing lists with Venn's diagrams. https://bioinfogp.cnb.csic.es/tools/venny/index.html.

[13] Xu Z, Zhou Y, Cao Y, Dinh TL, Wan J, Zhao M (2016). Identification of candidate biomarkers and analysis of prognostic values in ovarian cancer by integrated bioinformatics analysis. Med Oncol.

[14] Siegel RL, Miller KD, Jemal A (2016). Cancer statistics, 2016. CA Cancer J Clin.

[15] Krol J, Loedige I, Filipowicz W (2010). The widespread regulation of microRNA biogenesis, function and decay. Nat Rev Genet.

[16] Bartel DP (2004). MicroRNAs: genomics, biogenesis, mechanism, and function. Cell..

[17] Calin GA, Croce CM (2006). MicroRNA signatures in human cancers. Nat Rev Cancer.

[18] Madhavan D, Zucknick M, Wallwiener M, Cuk K, Modugno C, Scharpff M (2012). Circulating miRNAs as surrogate markers for circulating tumor cells and prognostic markers in metastatic breast cancer. Clin Cancer Res..

[19] Rao Q, Shen Q, Zhou H, Peng Y, Li J, Lin Z (2012). Aberrant microRNA expression in human cervical carcinomas. Med Oncol.

[20] Toiyama Y, Hur K, Tanaka K, Inoue Y, Kusunoki M, Boland CR (2014). Serum miR-200c is a novel prognostic and metastasis-predictive biomarker in patients with colorectal cancer. Ann Surg.

[21] Williams LV, Veliceasa D, Vinokour E, Volpert OV (2013). miR-200b inhibits prostate cancer EMT, growth and metastasis. PLoS One.

[22] Kurashige J, Kamohara H, Watanabe M, Hiyoshi Y, Iwatsuki M, Tanaka Y (2012). MicroRNA-200b regulates cell proliferation, invasion, and migration by directly targeting ZEB2 in gastric carcinoma. Ann Surg Oncol.

[23] Wong CM, Wei L, Au SL, Fan DN, Zhou Y, Tsang FH (2015). MiR-200b/200c/429 subfamily negatively regulates rho/ROCK signaling pathway to suppress hepatocellular carcinoma metastasis. Oncotarget..

[24] Dai Y, Xia W, Song T, Su X, Li J, Li S (2013). MicroRNA-200b is overexpressed in endometrial adenocarcinomas and enhances MMP2 activity by downregulating TIMP2 in human endometrial cancer cell line HEC-1A cells. Nucleic acid therapeutics.

[25] Antolin S, Calvo L, Blanco-Calvo M, Santiago MP, Lorenzo-Patino MJ, Haz-Conde M (2015). Circulating miR-200c and miR-141 and outcomes in patients with breast cancer. BMC Cancer.

[26] Song C, Liu LZ, Pei XQ, Liu X, Yang L, Ye F (2015). miR-200c inhibits breast cancer proliferation by targeting KRAS. Oncotarget..

[27] Lin HM, Castillo L, Mahon KL, Chiam K, Lee BY, Nguyen Q (2014). Circulating microRNAs are associated with docetaxel chemotherapy outcome in castration-resistant prostate cancer. Br J Cancer.

[28] Gao YC, Wu J (2015). MicroRNA-200c and microRNA-141 as potential diagnostic and prognostic biomarkers for ovarian cancer. Tumour Biol.

[29] Karaayvaz M, Zhang C, Liang S, Shroyer KR, Ju J (2012). Prognostic significance of miR-205 in endometrial cancer. PLoS One.

[30] Snowdon J, Zhang X, Childs T, Tron VA, Feilotter H (2011). The microRNA-200 family is upregulated in endometrial carcinoma. PLoS One.

[31] Han Y, Chen J, Zhao X, Liang C, Wang Y, Sun L (2011). MicroRNA expression signatures of bladder cancer revealed by deep sequencing. PLoS One.

[32] Nam EJ, Yoon H, Kim SW, Kim H, Kim YT, Kim JH (2008). MicroRNA expression profiles in serous ovarian carcinoma. Clin Cancer Res.

[33] Uhlmann S, Zhang JD, Schwager A, Mannsperger H, Riazalhosseini Y, Burmester S (2010). miR-200bc/429 cluster targets PLCgamma1 and differentially regulates proliferation and EGF-driven invasion than miR-200a/141 in breast cancer. Oncogene..

[34] Sun T, Wang C, Xing J, Wu D (2011). miR-429 modulates the expression of c-myc in human gastric carcinoma cells. Eur J Cancer.

[35] Brunet Vega A, Pericay C, Moya I, Ferrer A, Dotor E, Pisa A (2013). microRNA expression profile in stage III colorectal cancer: circulating miR-18a and miR-29a as promising biomarkers. Oncol Rep.

[36] Lu YB, Hu JJ, Sun WJ, Duan XH, Chen X (2015). Prognostic value of miR-141 downregulation in gastric cancer. Genet Mol Res.

[37] Wszolek MF, Rieger-Christ KM, Kenney PA, Gould JJ, Silva Neto B, Lavoie AK (2011). A MicroRNA expression profile defining the invasive bladder tumor phenotype. Urol Oncol.

[38] Wu CT, Lin WY, Chang YH, Lin PY, Chen WC, Chen MF (2015). DNMT1-dependent suppression of microRNA424 regulates tumor progression in human bladder cancer. Oncotarget..

[39] Zhou Y, An Q, Guo RX, Qiao YH, Li LX, Zhang XY (2017). miR424-5p functions as an anti-oncogene in cervical cancer cell growth by targeting KDM5B via the notch signaling pathway. Life Sci.

[40] Li Q, Qiu XM, Li QH, Wang XY, Li L, Xu M (2015). MicroRNA-424 may function as a tumor suppressor in endometrial carcinoma cells by targeting E2F7. Oncol Rep.

[41] Cai C, Chen QB, Han ZD, Zhang YQ, He HC, Chen JH (2015). miR-195 inhibits tumor progression by targeting RPS6KB1 in human prostate Cancer. Clin Cancer Res..

[42] Tsukamoto O, Miura K, Mishima H, Abe S, Kaneuchi M, Higashijima A (2014). Identification of endometrioid endometrial carcinoma-associated microRNAs in tissue and plasma. Gynecol Oncol.

[43] Wang R, Zhao N, Li S, Fang JH, Chen MX, Yang J (2013). MicroRNA-195 suppresses angiogenesis and metastasis of hepatocellular carcinoma by inhibiting the expression of VEGF, VAV2, and CDC42. Hepatology..

[44] Sauer K, Lehner CF (1995). The role of cyclin E in the regulation of entry into S phase. Progress Cell Cycle Res.

[45] Han Z, Zhang Y, Yang Q, Liu B, Wu J, Zhang Y (2015). miR-497 and miR-34a retard lung cancer growth by co-inhibiting cyclin E1 (CCNE1). Oncotarget..

[46] Liang Y, Gao H, Lin SY, Goss JA, Brunicardi FC, Li K (2010). siRNA-based targeting of cyclin E overexpression inhibits breast cancer cell growth and suppresses tumor development in breast cancer mouse model. PLoS One.

[47] Mao L, Ding J, Perdue A, Yang L, Zha Y, Ren M (2012). Cyclin E1 is a common target of BMI1 and MYCN and a prognostic marker for neuroblastoma progression. Oncogene..

[48] Nakayama N, Nakayama K, Shamima Y, Ishikawa M, Katagiri A, Iida K (2010). Gene amplification CCNE1 is related to poor survival and potential therapeutic target in ovarian cancer. Cancer..

[49] Hunt KK, Keyomarsi K (2005). Cyclin E as a prognostic and predictive marker in breast cancer. Semin Cancer Biol.

[50] Lopez-Beltran A, MacLennan GT, Montironi R (2006). Cyclin E as molecular marker in the management of breast cancer: a review. Anal Quant Cytol Histol.

[51] Zhang X, Hu S, Zhang X, Wang L, Zhang X, Yan B (2014). MicroRNA-7 arrests cell cycle in G1 phase by directly targeting CCNE1 in human hepatocellular carcinoma cells. Biochem Biophys Res Commun.

[52] Wang J, Xu G, Shen F, Kang Y (2014). miR-132 targeting cyclin E1 suppresses cell proliferation in osteosarcoma cells. Tumour Biol.

[53] Zubillaga-Guerrero MI, Alarcon-Romero Ldel C, Illades-Aguiar B, Flores-Alfaro E, Bermudez-Morales VH, Deas J (2015). MicroRNA miR-16-1 regulates CCNE1 (cyclin E1) gene expression in human cervical cancer cells. Int J Clin Exp Med.

[54] Matsushita R, Seki N, Chiyomaru T, Inoguchi S, Ishihara T, Goto Y (2015). Tumour-suppressive microRNA-144-5p directly targets CCNE1/2 as potential prognostic markers in bladder cancer. Br J Cancer.

[55] Day RS, McDade KK, Chandran UR, Lisovich A, Conrads TP, Hood BL (2011). Identifier mapping performance for integrating transcriptomics and proteomics experimental results. BMC bioinformatics.

[56] Ritchie ME, Phipson B, Wu D, Hu Y, Law CW, Shi W (2015). Limma powers differential expression analyses for RNA-sequencing and microarray studies. Nucleic Acids Res.

[57] Hardcastle TJ (2016). Generalized empirical Bayesian methods for discovery of differential data in high-throughput biology. Bioinformatics..

[58] Huang da W, Sherman BT, Lempicki RA (2009). Systematic and integrative analysis of large gene lists using DAVID bioinformatics resources. Nat Protoc.

[59] Szklarczyk D, Franceschini A, Wyder S, Forslund K, Heller D, Huerta-Cepas J (2015). STRING v10: protein-protein interaction networks, integrated over the tree of life. Nucleic Acids Res.

[60] Shannon P, Markiel A, Ozier O, Baliga NS, Wang JT, Ramage D (2003). Cytoscape: a software environment for integrated models of biomolecular interaction networks. Genome Res.

[61] Bader GD, Hogue CW (2003). An automated method for finding molecular complexes in large protein interaction networks. BMC bioinformatics.

[62] Xiao F, Zuo Z, Cai G, Kang S, Gao X, Li T (2009). miRecords: an integrated resource for microRNA-target interactions. Nucleic Acids Res.

[63] Li JH, et al. starBase v2.0: decoding miRNA-ceRNA, miRNA-ncRNA and protein-RNA interaction networks from large-scale CLIP-Seq data. Nucleic Acids Res. 2014;42:D92–7. 10.1093/nar/gkt1248 PMC3964941 24297251

[64] Anaya J. OncoLnc: linking TCGA survival data to mRNAs, miRNAs, and lncRNAs. Peer J Computer Science. 2016;2:e67.

