# Supplementary material for: Bioinformatic screening for candidate biomarkers and their prognostic values in endometrial cancer
Source: BMC Genet. 2020 Sep 22;21:113. doi: 10.1186/s12863-020-00898-4 (PMC7510080; doi:10.1186/s12863-020-00898-4)
Supplement: Supplementary file 1 — Additional file 1. Node-degree of interaction analysis of the 82 hub genes (Degree of interaction ≥10). [file 12863_2020_898_MOESM1_ESM.pdf]

Node-degree of interaction analysis of the 82 hub genes (Degree of interaction ≥ 10).

Node	Degree of interaction
CDK1	74
CCNA2	72
BUB1	68
CCNB1	67
CDC20	66
TOP2A	65
KIF11	64
CDCA8	63
CCNB2	63
KIF20A	60
NCAPG	60
NUSAP1	59
TTK	59
MELK	58
ASPM	57
UBE2C	57
BIRC5	56
MAD2L1	55
KIF15	53
CENPE	53
KIF23	53
CEP55	52
HJURP	51
CHEK1	50
CENPA	50
MCM10	49
FOXM1	48
NEK2	48
DTL	47
PRC1	47
PTTG1	47
SPAG5	46
EXO1	46
ESPL1	45
OIP5	44
TYMS	44
KIF14	43
CENPN	42
GINS2	41
MCM4	41
PLK4	40
CDC25C	40
CENPM	39
RAD54L	37
ECT2	35
CDT1	35
CDCA3	34
KIF18A	34
CKS2	34
SHCBP1	33
RAD51	33
DEPDC1	31
KIF18B	28
GTSE1	28
ERCC6L	26
CDCA2	21
CKAP2L	21
CDC25A	21
ATAD2	20
MYC	20
E2F8	18
EZH2	18
TK1	18
GNG7	17
STIL	16
CCNF	16
E2F1	15
BLM	14
SAA1	13
CDCA7	13
CXCL8	13
TIMELESS	13
PKMYT1	13
CCNE1	13
FGF2	12
APOE	12
FBN1	11

SERPINA1	11
ORC6	11
GABBR1	10
SMARCA4	10
GAPDH	10
