# Supplementary material for: Bioinformatic screening for candidate biomarkers and their prognostic values in endometrial cancer
Source: BMC Genet. 2020 Sep 22;21:113. doi: 10.1186/s12863-020-00898-4 (PMC7510080; doi:10.1186/s12863-020-00898-4)
Supplement: Supplementary file 2 — Additional file 2. Correlation between differentially expressed miRNAs and target genes in patients with endometrial cancer (Data source: starBase v2.0 project). [file 12863_2020_898_MOESM2_ESM.pdf]

Correlation between differentially expressed miRNAs and target genes in patients with endometrial cancer (Data source: starBase v2.0 project).

miRNA	Target gene	r	P-value	FDR
hsa-miR-100	CDC25A	-0.30159	1.01E-04	1.39E-03
hsa-miR-376c	DTL	-0.2903	1.87E-04	2.29E-03
hsa-miR-653	FGF2	-0.27245	4.72E-04	4.79E-03
hsa-miR-653	TFPI	-0.27922	3.35E-04	3.64E-03
hsa-miR-653	KLF9	-0.18617	1.81E-02	7.84E-02
hsa-miR-653	ADAMTS5	-0.20703	8.41E-03	4.45E-02
hsa-miR-653	EDNRA	-0.22888	3.50E-03	2.29E-02
hsa-miR-653	DIXDC1	-0.18902	1.63E-02	7.28E-02
hsa-miR-653	SPON1	-0.2516	1.28E-03	1.05E-02
hsa-miR-135b	NR3C2	-0.28211	2.88E-04	3.24E-03
hsa-miR-135b	RECK	-0.49029	4.07E-11	4.72E-09
hsa-miR-135b	KLF4	-0.29339	1.59E-04	2.00E-03
hsa-miR-135b	PALMD	-0.36711	1.67E-06	4.65E-05
hsa-miR-135b	COL5A1	-0.18939	1.61E-02	7.21E-02
hsa-miR-135b	PDE7B	-0.31731	4.11E-05	6.66E-04
hsa-miR-200c	MAMDC2	-0.61104	7.46E-18	3.94E-15
hsa-miR-200c	CYP1B1	-0.53439	2.86E-13	5.73E-11
hsa-miR-200c	ZNF302	-0.20162	1.03E-02	5.19E-02
hsa-miR-200c	RECK	-0.68965	4.68E-24	1.16E-20
hsa-miR-200c	KLF4	-0.45001	2.11E-09	1.54E-07
hsa-miR-200c	BNC2	-0.65375	5.39E-21	5.73E-18
hsa-miR-200c	PLCL1	-0.66453	7.20E-22	9.48E-19
hsa-miR-200c	LCA5	-0.34311	8.37E-06	1.78E-04
hsa-miR-200c	EDNRA	-0.68424	1.45E-23	2.93E-20
hsa-miR-200c	DZIP1	-0.47313	2.33E-10	2.21E-08
hsa-miR-200c	NR3C1	-0.47958	1.22E-10	1.26E-08
hsa-miR-200c	SPG20	-0.42485	1.94E-08	1.05E-06
hsa-miR-200c	AMOTL2	-0.39366	2.39E-07	8.96E-06
hsa-miR-200c	ZEB2	-0.65337	5.78E-21	6.07E-18
hsa-miR-200c	KCTD15	-0.38574	4.34E-07	1.49E-05
hsa-miR-200c	PDE7B	-0.6112	7.27E-18	3.86E-15
hsa-miR-200c	ZBTB38	-0.4857	6.55E-11	7.20E-09
hsa-miR-200c	DIXDC1	-0.67611	7.52E-23	1.25E-19
hsa-miR-200c	KLF10	-0.35937	2.85E-06	7.27E-05
hsa-miR-200c	PPM1F	-0.67147	1.88E-22	2.84E-19
hsa-miR-200c	MBNL2	-0.52117	1.36E-12	2.33E-10

hsa-miR-200c	CLIC4	-0.60419	2.15E-17	1.04E-14
hsa-miR-96	RECK	-0.63216	2.40E-19	1.77E-16
hsa-miR-96	IRS1	-0.43854	5.93E-09	3.78E-07
hsa-miR-96	BNC2	-0.66914	2.96E-22	4.28E-19
hsa-miR-96	AMOTL2	-0.41952	3.04E-08	1.54E-06
hsa-miR-96	NR4A3	-0.41974	2.98E-08	1.51E-06
hsa-miR-96	PDE7B	-0.61567	3.59E-18	2.03E-15
hsa-miR-96	DIXDC1	-0.64346	3.42E-20	3.04E-17
hsa-miR-96	PPM1F	-0.60083	3.59E-17	1.64E-14
hsa-miR-96	MBNL2	-0.51843	1.87E-12	3.07E-10
hsa-miR-429	ZNF662	-0.23463	2.74E-03	1.90E-02
hsa-miR-429	MAMDC2	-0.65825	2.35E-21	2.68E-18
hsa-miR-429	CYP1B1	-0.53786	1.87E-13	3.92E-11
hsa-miR-429	ZNF302	-0.27454	4.24E-04	4.41E-03
hsa-miR-429	RECK	-0.68806	6.55E-24	1.49E-20
hsa-miR-429	KLF4	-0.43547	7.78E-09	4.79E-07
hsa-miR-429	BNC2	-0.61497	4.01E-18	2.25E-15
hsa-miR-429	PLCL1	-0.66827	3.51E-22	4.91E-19
hsa-miR-429	KLF9	-0.55014	4.05E-14	9.87E-12
hsa-miR-429	EDNRA	-0.63255	2.25E-19	1.67E-16
hsa-miR-429	DZIP1	-0.47232	2.52E-10	2.37E-08
hsa-miR-429	NR3C1	-0.52214	1.22E-12	2.11E-10
hsa-miR-429	AMOTL2	-0.38854	3.52E-07	1.25E-05
hsa-miR-429	ZEB2	-0.63621	1.21E-19	9.37E-17
hsa-miR-429	KCTD15	-0.42602	1.76E-08	9.63E-07
hsa-miR-429	ZBTB38	-0.45053	2.01E-09	1.48E-07
hsa-miR-429	DIXDC1	-0.6784	4.75E-23	8.21E-20
hsa-miR-429	KLF10	-0.32158	3.19E-05	5.41E-04
hsa-miR-429	PPM1F	-0.6927	2.46E-24	6.52E-21
hsa-miR-429	CLIC4	-0.53733	2.00E-13	4.15E-11
hsa-miR-141	OLFM1	-0.45291	1.61E-09	1.22E-07
hsa-miR-141	ZNF302	-0.18682	1.76E-02	7.71E-02
hsa-miR-141	TLE4	-0.43342	9.30E-09	5.57E-07
hsa-miR-141	C1orf21	-0.42973	1.28E-08	7.35E-07
hsa-miR-141	KLF9	-0.54028	1.39E-13	3.00E-11
hsa-miR-141	NR3C1	-0.4588	9.31E-10	7.52E-08
hsa-miR-141	ZEB2	-0.62042	1.67E-18	1.02E-15
hsa-miR-141	COL15A1	-0.54532	7.46E-14	1.70E-11
hsa-miR-141	HOXA11	-0.21814	5.44E-03	3.21E-02
hsa-miR-141	DIXDC1	-0.67916	4.08E-23	7.21E-20

hsa-miR-141	LPP	-0.38254	5.50E-07	1.82E-05
hsa-miR-200a	OLFM1	-0.48111	1.05E-10	1.10E-08
hsa-miR-200a	ZNF302	-0.19774	1.19E-02	5.78E-02
hsa-miR-200a	C1orf21	-0.43953	5.44E-09	3.50E-07
hsa-miR-200a	NR3C1	-0.46959	3.30E-10	3.01E-08
hsa-miR-200a	ZEB2	-0.61701	2.90E-18	1.69E-15
hsa-miR-200a	COL15A1	-0.50654	7.10E-12	1.01E-09
hsa-miR-200a	DIXDC1	-0.66461	7.08E-22	9.36E-19
hsa-miR-200a	LPP	-0.36651	1.74E-06	4.82E-05
hsa-miR-182	RECK	-0.67319	1.34E-22	2.12E-19
hsa-miR-182	BNC2	-0.69315	2.23E-24	6.00E-21
hsa-miR-182	FBN1	-0.60013	3.98E-17	1.80E-14
hsa-miR-182	AMOTL2	-0.44388	3.69E-09	2.50E-07
hsa-miR-182	NR4A3	-0.43926	5.57E-09	3.57E-07
hsa-miR-182	PDE7B	-0.63639	1.17E-19	9.17E-17
hsa-miR-182	DIXDC1	-0.66215	1.13E-21	1.42E-18
hsa-miR-182	MBNL2	-0.56894	3.44E-15	1.04E-12
hsa-miR-183	TUB	-0.51299	3.46E-12	5.32E-10
hsa-miR-183	AKAP12	-0.50237	1.12E-11	1.51E-09
hsa-miR-183	TLE4	-0.53533	2.55E-13	5.16E-11
hsa-miR-183	IRS1	-0.49923	1.57E-11	2.04E-09
hsa-miR-183	BNC2	-0.70905	6.68E-26	2.73E-22
hsa-miR-183	NR4A2	-0.21781	5.51E-03	3.24E-02
hsa-miR-183	NR3C1	-0.59741	6.00E-17	2.63E-14
hsa-miR-27b	PNKD	-0.17758	2.42E-02	9.70E-02
hsa-miR-27b	STRBP	-0.15855	4.46E-02	1.50E-01
hsa-miR-27b	NEK2	-0.177	2.47E-02	9.83E-02
hsa-miR-29a	F11R	-0.25472	1.11E-03	9.41E-03
hsa-miR-29a	HMGCR	-0.22908	3.47E-03	2.27E-02
hsa-miR-29a	ENTPD7	-0.29038	1.87E-04	2.28E-03
hsa-miR-133b	STC2	-0.2241	4.27E-03	2.66E-02
hsa-miR-143	MYO6	-0.29866	1.19E-04	1.59E-03
hsa-miR-143	SIX4	-0.37194	1.19E-06	3.48E-05
hsa-miR-143	EME1	-0.3681	1.56E-06	4.38E-05
hsa-miR-143	HK2	-0.33768	1.18E-05	2.38E-04
hsa-miR-145	MYO6	-0.19803	1.18E-02	5.73E-02
hsa-miR-145	F11R	-0.42622	1.73E-08	9.49E-07
hsa-miR-145	RBM47	-0.31029	6.18E-05	9.31E-04
hsa-miR-195	WWC1	-0.29395	1.54E-04	1.96E-03
hsa-miR-195	PSAT1	-0.44755	2.64E-09	1.86E-07

hsa-miR-195	VAMP8	-0.25436	1.13E-03	9.54E-03
hsa-miR-195	SIX4	-0.15662	4.73E-02	1.56E-01
hsa-miR-195	MAP7	-0.37255	1.13E-06	3.36E-05
hsa-miR-195	CCNE1	-0.4651	5.10E-10	4.41E-08
hsa-miR-195	CHEK1	-0.31279	5.35E-05	8.28E-04
hsa-miR-195	LAMP3	-0.27888	3.41E-04	3.69E-03
hsa-miR-195	KIF23	-0.43825	6.09E-09	3.87E-07
hsa-miR-195	CDC25A	-0.38821	3.61E-07	1.27E-05
hsa-miR-200b	ZNF662	-0.24689	1.59E-03	1.25E-02
hsa-miR-200b	MAMDC2	-0.65154	8.06E-21	8.09E-18
hsa-miR-200b	RECK	-0.70905	6.68E-26	2.76E-22
hsa-miR-200b	KLF4	-0.42963	1.29E-08	7.40E-07
hsa-miR-200b	BNC2	-0.63153	2.67E-19	1.94E-16
hsa-miR-200b	PLCL1	-0.68213	2.23E-23	4.31E-20
hsa-miR-200b	LCA5	-0.34934	5.57E-06	1.27E-04
hsa-miR-200b	KLF9	-0.55255	2.98E-14	7.50E-12
hsa-miR-200b	EDNRA	-0.6654	6.09E-22	8.15E-19
hsa-miR-200b	NR3C1	-0.51945	1.66E-12	2.78E-10
hsa-miR-200b	SPG20	-0.42241	2.39E-08	1.25E-06
hsa-miR-200b	AMOTL2	-0.36278	2.25E-06	5.99E-05
hsa-miR-200b	ZEB2	-0.66269	1.02E-21	1.30E-18
hsa-miR-200b	KCTD15	-0.43494	8.15E-09	4.97E-07
hsa-miR-200b	PDE7B	-0.45295	1.61E-09	1.21E-07
hsa-miR-200b	ZBTB38	-0.45295	1.61E-09	1.21E-07
hsa-miR-200b	DIXDC1	-0.67889	4.31E-23	7.55E-20
hsa-miR-200b	KLF10	-0.29319	1.60E-04	2.02E-03
hsa-miR-200b	PPM1F	-0.72196	3.23E-27	2.01E-23
hsa-miR-200b	MBNL2	-0.51713	2.17E-12	3.51E-10
hsa-miR-200b	CLIC4	-0.54419	8.58E-14	1.94E-11
hsa-miR-379	MYO10	-0.26106	8.23E-04	7.44E-03
hsa-miR-379	CCNB1	-0.21339	6.57E-03	3.70E-02
hsa-miR-381	CCNA2	-0.23237	3.02E-03	2.04E-02
hsa-miR-381	KIF11	-0.27384	4.40E-04	4.53E-03
hsa-miR-411	RAD51	-0.19795	1.18E-02	5.74E-02
hsa-miR-411	DTL	-0.26921	5.54E-04	5.44E-03
hsa-miR-424	PSAT1	-0.24388	1.82E-03	1.39E-02
hsa-miR-424	SIX4	-0.19492	1.32E-02	6.23E-02
hsa-miR-424	MAP7	-0.23747	2.42E-03	1.73E-02
hsa-miR-424	CCNE1	-0.33075	1.82E-05	3.41E-04
hsa-miR-424	CHEK1	-0.24735	1.56E-03	1.23E-02

hsa-miR-424	LAMP3	-0.19535	1.30E-02	6.16E-02
hsa-miR-424	KIF23	-0.42687	1.63E-08	9.04E-07
hsa-miR-424	CDC25A	-0.31268	5.39E-05	8.33E-04
hsa-miR-424	CEP55	-0.35167	4.77E-06	1.12E-04

miRNA or miR, microRNA; FDR, false discovery rate.
